# Peer tutoring in a medical school: perceptions of tutors and tutees

**DOI:** 10.1186/s12909-016-0589-1

**Published:** 2016-03-08

**Authors:** Annette Burgess, Tim Dornan, Antonia J. Clarke, Audrey Menezes, Craig Mellis

**Affiliations:** Education Office, Sydney Medical School, The University of Sydney, Edward Ford Building A27, Sydney, NSW 2006 Australia; Queen’s University Belfast, Belfast, UK; Maastricht University, Maastricht, The Netherlands; Royal Prince Alfred Hospital, Camperdown, NSW Australia; Hornsby Hospital, Hornsby, NSW Australia; Sydney Medical School-Central, Sydney Medical School, The University of Sydney, Sydney, NSW Australia

## Abstract

**Background:**

Peer tutoring has been described as “people from similar social groupings who are not professional teachers helping each other to learn and learning themselves by teaching”. Peer tutoring is well accepted as a source of support in many medical curricula, where participation and learning involve a process of socialisation. Peer tutoring can ease the transition of the junior students from the university class environment to the hospital workplace. In this paper, we apply the Experienced Based Learning (ExBL) model to explore medical students’ perceptions of their experience of taking part in a newly established peer tutoring program at a hospital based clinical school.

**Methods:**

In 2014, all students at Sydney Medical School – Central, located at Royal Prince Alfred Hospital were invited to voluntarily participate in the peer tutoring program. Year 3 students (*n* = 46) were invited to act as tutors for Year 1 students (*n* = 50), and Year 4 students (*n* = 60) were invited to act as tutors for Year 2 students (*n* = 51). Similarly, the ‘tutees’ were invited to take part on a voluntary basis. Students were invited to attend focus groups, which were held at the end of the program. Framework analysis was used to code and categorise data into themes.

**Results:**

In total, 108/207 (52 %) students participated in the program. A total of 42/106 (40 %) of Year 3 and 4 students took part as tutors; and of 66/101 (65 %) of Year 1 and 2 students took part as tutees. Five focus groups were held, with 50/108 (46 %) of students voluntarily participating. Senior students (tutors) valued the opportunity to practice and improve their medical knowledge and teaching skills. Junior students (tutees) valued the opportunity for additional practice and patient interaction, within a relaxed, small group learning environment.

**Conclusion:**

Students perceived the peer tutoring program as affording opportunities not otherwise available within the curriculum. The peer teaching program provided a framework within the medical curriculum for senior students to practice and improve their medical knowledge and teaching skills. Concurrently, junior students were provided with a valuable learning experience that they reported as being qualitatively different to traditional teaching by faculty.

## Background

Medical students acquire knowledge, skills and attitudes required for medical practice largely through curricular activities at hospital based clinical schools. An integrated approach to learning and teaching, that orientates students to the cultural and social aspects of medicine, helps students to shape their professional values as they prepare for medical practice [1]. Junior medical students new to the hospital environment often find their new learning experiences, activities and assessments challenging. As such, they need to be offered meaningful opportunities to participate [2], as it is only through participation that new practices can be learnt, and progressively, new tasks can be undertaken. Although the majority of medical programs are now vertically integrated, and provide early clinical exposure within the hospital setting, availability of hospital clinicians for teaching is well recognised as an issue within medical education [3, 4]. This highlights the importance of the provision of additional support, such as peer assisted learning activities, for junior students, who are initially on the boundaries of the new community they are entering.

Described as “people from similar social groupings who are not professional teachers helping each other to learn and learning themselves by teaching” [5], peer assisted learning can help provide additional support for students, and address specific gaps within the curriculum [6–8]. Various forms of peer assisted learning are well accepted as a source of support in many medical curricula, where participation and learning involve a process of socialisation. Through *peer tutoring* activities, student interaction is facilitated within a formal, professional context, easing the transition of the junior students from the university class environment to the hospital workplace. Student peers have a common interest in studying medicine, and their social and cognitive congruence provide a unique invitational quality. Senior students are able to offer junior students opportunities to familiarise themselves with the language, tasks and processes of the new environment. Concurrently, the peer tutoring process contributes to the development of the professional identity of senior students who, as medical practitioners in the near future, will be expected to teach and assess peers. Although junior students are not given the scope to practice their own teaching and assessment skills, they may develop an appreciation of their own future roles and responsibilities as senior members of the student community.

Theories informing educational practice offer valuable lenses to observe, design and analyse teaching and learning [9]. Dornan and colleagues (2014) have suggested a transferrable blueprint for clerkship education [10]. The Experienced Based Learning (ExBL) model proposes that medical student learning outcomes are acquired through participation in activities within authentic learning environments [10]. As Dornan and colleagues (2014) state, “clerkship education does not just happen” [10]. Rather, students learn when resources are creatively used to construct ideal conditions for learning [10]. That is, their learning is largely dependent upon the affective, pedagogic and organisational support facilitated by their medical school and hospital workplace.

This study took place in a clinical school based at a large metropolitan teaching hospital, where students are afforded the opportunity to participate in newly established Peer Tutoring (PT) program. In this context, we sought to explore students’ perceptions of their experience, both as peer tutors and tutees, in taking part in this PT program.

The purpose of this study was to demonstrate the implementation and outcomes of a student led peer tutoring program within a medical school, so that staff in other medical schools might benefit from our experience. By sharing our experience, others might in turn consider the support mechanisms required, when designing or improving their own peer assisted learning programs.

In this paper, we utilise the ExBL model to explore students’ experience of participation in this newly established peer tutoring program. Although other work place models were considered, we chose the ExBL model because of its relevance. Specifically designed for medical students, this model places an emphasis on the *support* structures required for implementation of educational activities within the clinical environment. These are areas that are often not explicitly addressed in work place models.

Our research question was “How does the *support* afforded within a new peer tutoring program at a hospital based clinical school assist students, both as peer tutors and tutees, in development of their knowledge, skills and attitudes?”

## Methods

### Conceptual orientation

We utilised the ExBL model to explore students’ experiences of participation in a newly established peer tutoring program at a large metropolitan teaching hospital. According to the ExBL model, students’ learning processes are fostered by three key areas of support:

1. *Organisational support* ensures that the learning experience sits appropriately within the curriculum, with opportunities to participate in practice.
2. *Pedagogic support* is provided by teachers in the workplace setting, including mentors, supervisors, role models, as well as sources of informal support.
3. *Affective support* is provided by a warm an inclusive learning environment.

### Recruitment and sampling

In 2014, a formal peer tutoring program was established at one metropolitan “home clinical school”, Central Clinical School (CCS), located at Royal Prince Alfred Hospital, Sydney. In total, Sydney Medical School has six metropolitan home clinical schools, with a total student population of 1,200. All students within CCS (*n* = 207) were invited by email to take part on a voluntary basis. Year 3 students (total = 46) were invited to act as tutors for Year 1 students (total = 50), and all Year 4 students (total = 60) as tutors for Year 2 students (total = 51). The study took place over the course of the 2014 academic year, and participation was voluntary.

### Context

#### Traditional workplace learning methods

The University of Sydney offers a 4 year, graduate entry medical program. First and second year students enrolled in the Sydney Medical Program (SMP) attend University main campus 4 days per week. One day per week, students attend their “home clinical school”. Home clinical schools are based at one of six metropolitan teaching hospitals. There, on a weekly basis, students take part in small group, bedside clinical tutorials. Students attend 1.5 h of history taking, and 1.5 h of physical examination skills tutorials. Each group has one tutor, who is either a senior specialist (consultant), or a registrar (house staff), or a general practitioner. Students are also encouraged to seek experience pro-actively, and practice these skills with patients on the wards at the hospital.

#### Peer tutoring intervention

The peer tutoring activity augmented the students’ traditional work place learning activities within the hospital. That is, it provided students with meaningful, supervised opportunities to gain practical experience within the real workplace setting, and mirror their future professional roles as medical practitioners. The content of the PT program was aligned with the students’ existing curriculum, that is, the topics taught were aligned with each ‘block’ of teaching (from block 1 to block 8), which included:

- Year 1: 1) Musculoskeletal 2) Respiratory 3) Haematology, 4) Cardiovascular
- Year 2 : 5) Neurosciences 6) Endocrinology 7) Gastroenterology 8) Oncology and Palliative care.

#### Tutorial design and format

The PT program was designed, organised and led by two Year 4 students. Central Clinical staff were involved in organisation in terms of email distribution, room bookings, and endorsement of the program. Three to four tutees were assigned to a pair of tutors. Tutors were allocated in pairs to ensure that at least one tutor was available for each tutorial. It was suggested that tutorials occur at a minimum of fortnightly, but more frequently if desired.

Tutorials were approximately one hour long, covering clinically relevant content. Tutorials were designed to supplement existing teaching in order to enhance the tutees’ knowledge base. The delivery of tutorials was intended to be interactive, with the ideal tutorial having 20 min of briefing/explanation of a concept, 20 min of application of the content/on the wards, and 20 min debriefing/discussion.

#### Preparation for tutorials

All participants (tutors and tutees) were provided with a one-hour information session facilitated by the two student leaders, detailing the objectives of the program, the logistics of organising tutor groups, and the format of the tutorials. Tutees were responsible for identifying a relevant curriculum topic within the current teaching block, and notifying their tutor a few days in advance of the tutorial, allowing tutors adequate preparation time. Prior to each tutorial, tutors were required to create a one-page handout. Each handout was reviewed by a Junior Medical Officer at Royal Prince Alfred Hospital to ensure accuracy. Handouts were uploaded to a shared online folder, accessible by all participants in the program, allowing students to benefit from other tutorials as well as their own.

### Focus groups

All participants were invited to attend a focus group at the completion of the PT program. Focus groups were held in order to provide a deep understanding and more complex picture of students’ perceptions. The focus groups were held at the hospital and each was facilitated by one of three authors, and ran for approximately 1 h each in length. Focus groups for tutors and tutees were held separately so that participants could speak freely regarding their experience. The focus group questions specifically focused on the experiences of students during their hospital based peer tutorials. For example, “Did you develop any sense of community by working with other year groups?”.

### Data analysis

Focus group data were recorded and transcribed verbatim. A thematic analysis of the qualitative data was carried out using Framework Analysis [11]. ExBL model was used as a conceptual framework for the paper and subsequently a thematic framework was developed and applied to a portion of the dataset by the first author to pilot the coding framework. Coding focused on the socio-cultural experiences and interactions of the peer tutors and tutees. After independently reading the same data sample, three of the authors met to discuss and develop this coding framework, and to assess any emerging themes that would extend the analysis. Subsequently, the first author coded all of the data, in order to identify recurrent themes and subthemes in the data [12].

### Ethical considerations

Ethics approval was obtained from The University of Sydney Human Research Ethics Committee. All participants were reassured that data was strictly de-identified to protect participant privacy. All participants gave their written consent to participate in the study.

## Results

In total, 108/207 (52 %) of all CCS students participated in the program. Of the participating tutors, 19/42 (45 %) were Year 3 students and 23/42 (55 %) were Year 4 students. Of the participating tutees, 34/66 (52 %) were Year 1 students and 32/66 (48 %) were Year 2 students.

### Focus group results

A total of five focus groups were held, with 50/108 (46 %) students voluntarily attending. Of these focus group participants, 21/50 (42 %) were tutors, and 29/50 (58 %) were tutees.

We conducted two focus groups with the tutors, with 10 participants in the first, and 11 participants in the second group. We conducted three focus groups with the tutees, with 10 participants in the first, 10 participants in the second, and 9 participants in the third.

The results are presented using the conceptual framework of the Experienced Based Learning model (Dornan et al., 2104). The theme of Organisational support is illustrated in Table 1. *Organisational support* ensures that the learning experience sits appropriately within the curriculum, with opportunities to participate in practice. Although participants found small group teaching effective, and found it beneficial to have some input into the content, they would have appreciated greater input from the School staff. The theme of Pedagogic support is illustrated in Table 2. *Pedagogic support* is provided by teachers, mentors and role models in the workplace setting. Tutors reported development of competence in both content and teaching skills, and a sense of development of professional identity. Tutees felt their knowledge was reinforced by the tutorials, and that teaching by peers provided clear explanations that may otherwise be lacking with more senior teachers. Affective support is illustrated in Table 3*. Affective support* is provided a warm an inclusive learning environment. Participants perceived that the Peer Tutoring program provided a formal, but inclusive environment. Tutees found tutors to be approachable, and tutors enjoyed assisting junior peers in their learning.

**Table 1:** Participants’ responses to questions regarding their perceptions of their experiences that related to “Organisational support”

Organisational support ensures that the learning experience sits appropriately within the curriculum, with opportunities to participate in practice.	
Tutee responses	
*Tutee input into the curriculum topics*	*“It was an advantage that the free form of not having anything really set in stone. If we need to practice our respiratory examination, can we do this next week, then we’d ask to do an ABGs next week. So it was just what we thought we needed to learn, we’d ask about and then we’d talk about other things, using their experience”.*
*Opportunities to see patients*	*“I loved going out to the wards to see patients…. to have two or three people in a group after the six made it a little bit more intimate and we actually got to do more hands on”.*
	*“Having two tutors definitely maximized the number of sessions we did have”.*
*Tutors provided appropriate information and resources targeted at the tutees’ needs*	*“Our tutors gave us a lot of references and did a PowerPoint presentation and provided us with resources and then backup what they taught us, which was great. They went over everything that was exam relevant. ..they gave us summaries of OSCE stations”.*
*Additional monitoring and quality assurance and reward system required by Clinical School staff*	*“If they sign up, they should actually do it. You need to make sure they’re coming”.*
	*“The teaching should be evaluated, and that can be part of the tutors’ portfolio. That might be an incentive and also provide quality control.”*
	*“When they’re highly regarded as teachers, there should be some kind of a reference given or something like that”.*
Tutor responses	
*Effectiveness of small group teaching*	*“With very small groups of two to three, it was different to groups of six people. They felt like they were getting a lot more, sort of one on one. Even if they went on the wards, they felt like they were given all the attention which was nice. Also, it didn’t feel awkward like a whole room of people standing around one patient”.*
*Creation of resources targeted at tutees' needs*	*“I created a shoulder exam sheet and did a couple of powerpoint slides. I used the ones out of the drop box because the intern had checked it”.*
	*“I would screen shot something form Talley’s and use that as a talking point.”*
*Increased organisation and access to resources*	*“It would have been nice to have everything on the Dropbox because that was the original intention, is that if the tutor wrote up the summary then it was going to be posted out to the Dropbox so that all groups had access to it, but I don’t think that ended up happening”.*

**Table 2:** Participants’ responses to questions regarding their perceptions of their experiences that related to “Pedagogic support”

Pedagogic support is provided by teachers in the workplace setting, including mentors, supervisors, role models, as well as sources of informal support.	
Tutee responses	
*Knowledge for tutees was reinforced by the tutorials*	“*For me it was mostly reinforcement of what we were given in lectures and PBLs. We didn’t cover any new material but it brought everything together, they contextualised it in a way that they understood it to approach the exams”.*
*Tutees felt the tutors were enthusiastic about teaching*	*“One time the topic we were requesting was AVGs and our tutor actually then brought along whatever they call them, but he was basically like, let’s just take each other’s arterial blood. We each stuck a needle in someone and only one of us actually got any blood out. We had a procedural skills session on cannulation just recently. The next time we will encounter it is in an OSCE station or when you’re a resident. You don’t get to go over it again”.*
*Tutees found their peer tutors provided appropriate explanations and a clinical relevance to what was being taught, and also gave the necessary time to the tutees.*	“So it's really good to have the peer tutors - it is clinically based but also adding in *why you do certain things. Our tutors have more time to kind of go, “Well, this is why you're looking for that, this is why you're doing this,”*
	*“A lot of the physicians and the specialists will say, “Oh, yeah, there's this, and you can read about that later.” And so this is an opportunity to actually discuss those issues, rather than just, be expected to go read the whole textbook in a week and, you know, do that for every – every topic. So it was good to be able to get concise points, um, and have it still be based on credible experience”.*
*Tutees perceived tutors to be aware of the level of teaching needed*	*“I feel like when we have tutorials on the wards, the professors and consultants who teach us, teach us more than what we're supposed to know. They don't teach us at all what we're supposed to know for the exam. When we do tutoring with those a few years above us, they know exactly which point we need to know”.*
Tutor responses	
*Tutors felt that their recent previous experience allowed them to support their junior peers*	*“We know quite well the depth that they are going to need to know things. And we know what they have coming up… we’ve been through the OSCEs so we can teach them in a way that is the most relevant to them, because we’ve been there only a year or two before – we know.”*
*By tutoring, students realised how much knowledge they have gained as senior students, and their confidence to teach was increased.*	*“If I was asked a question, I’d know quite a lot of information, whereas I was concerned that maybe I wouldn’t really know a lot. It gave me a lot more confidence that I have something worthwhile to teach people who are a couple of years younger. It made me more likely to sign up for teaching in the future”*
*Tutors developed an appreciation for teaching as part of their future professional identity*	*“It’s good experience for the future. If other teaching opportunities come up, we will have already had experience teaching medical students, so it’s something good for* our own further progress as teachers”.

**Table 3:** Participants’ responses to questions regarding their perceptions of their experiences that related to “Affective support”

Affective support is provided a warm an inclusive learning environment.	
Tutee responses	
*The peer tutoring program provided a formal means for students to interact*	*“It's nice, as a first year, to go to an organised event outside RPA, and to walk in and know people from other years….and actually be able to have conversations.. to actually know them by name”.*
	*“Last year my only interaction with third and fourth years was doing our mock OSCE. This was a way to get to know the older years”.*
*Tutees perceived tutors to be proactive and welcoming*	*“Tutors were getting in contact with their group, saying, do you want to have extra sessions, is there anything else I can help you out with? Just being proactive about that, is really good”.*
*Tutors were approachable*	*“I felt really comfortable asking my tutor anything. And he'd reply happily so it's cool”.*
*Students felt comfortable asking their senior peers questions*	*“You could ask them questions second years can’t answer because we don’t know. But you don’t want to ask an intern or a doctor that question because that seems inappropriate. So it’s a perfect median-like way and they can just give you a straight answer. Like picking your rotations for third year or things like that, they give you tips. That was really useful”.*
Tutor responses	
*A sense of community was fostered through the peer tutoring program*	*“It's kind of nice to foster a little bit of community throughout the year levels”.*
*Tutors enjoyed teaching and developed confidence to teach in the future*	*“I definitely would now be much more happy to teach people because I had a good time and it wasn’t as stressful as I thought it would be”.*
	*“It was a real pleasure to teach people who were all there voluntarily and really wanted to learn”.*

## Discussion

This study sought to explore students’ perceptions of taking part in the peer tutoring program. As reflected in our results, students perceived the Peer Tutoring program afforded opportunities not otherwise available within the curriculum. The learning environment was enriched by a framework that allowed tutors to practice and improve their medical knowledge and professionalism skills in teaching and communication [13]. Additionally, tutees were provided with a valuable learning experience that was qualitatively different to traditional teaching by faculty [5]. Although there is some overlap between categories, the students’ experience of the peer tutoring program can be illustrated within three key areas: Organisational support, Pedagogic support, and Affective support.

### Organisational support

Organisational support ensures the students’ learning experience aligns with the curriculum outcomes, and provides opportunities to participate in practice [10]. Commitment, order and engagement from both peer tutors and tutees were achieved through careful planning, preparation and set guidelines [14]. Clear guidelines ensured that the *content* being taught was appropriately aligned with the curriculum, and with tutees’ needs. Learning is context dependent [15], and activities that took place at the bedside with multiple opportunities for patient interaction, provided authentic experiences for the students. Both tutors and tutees felt having only two to three students per group promoted this interaction. Feedback indicated that additional support from the Clinical School was required, particularly in terms of quality assurance. Tutees felt that formal recognition of tutor participation would enhance the program.

### Pedagogic support

Pedagogic support includes support for practice-based learning, which is provided in the workplace by teachers [10]. The social and cognitive congruence of senior and junior peers afforded a unique richness to learning [13]. Although the learning trajectory of each medical student is unique [16], compared to faculty, junior students perceived that their senior peers had a greater awareness of the curriculum and assessment requirements, and their capabilities. Senior students were able to make explicit aspects of the learning process that were not always explained by senior clinical teachers [15, 17]. During peer tutoring activities, both senior and junior peers were dependent upon each other’s relevant experiences and shared resources. Rather than being individually constructed, knowledge and skills were socially constructed [18].

By allowing senior students to act as tutors of their junior peers, their responsibilities were aligned with their abilities, and competence was developed [14]. Tutors became active participants in the development of their own professional identities. A focus on professional identity formation is highly relevant in contemporary medical education, where medical students may have a number of different roles [19]. The PT program emphasised the professional expectations of students as medical graduates. Senior students undertook meaningful educational tasks that required preparation, and mirrored the roles they will assume as junior medical officers. Internationally, teaching skills are increasingly recognised by medical schools and medical councils as requisite graduate competencies [20]. The PT program enabled these expectations to be met by providing students with opportunities to develop their teaching and communication skills. By participating in PT, students recognised the importance of these skills within medicine and associated these attributes with their identity as medical student and future medical practitioners [20].

### Affective support

Affective support is that which provides a warm and inclusive learning environment [10]. A sense of belonging is fostered when students feel they are being treated as members of one community, with similar goals and purpose [21, 22]. The learning environment afforded by the tutors promoted supportive interactions between the tutors and tutees that fostered confidence for both groups of students. Tutors gained confidence in their own knowledge, and in their teaching skills.

The actions of tutors are important in shaping the culture of the learning environment, and help to create a relaxed, collegial atmosphere [15]. Through the peer tutoring program, a supportive and professional culture evolved within the student community. Tutors not only took part to reinforce their own medical knowledge, and develop their teaching skills, but saw participation as a way to *“foster ….community throughout the year level”* and found it *“a pleasure to teach people”*, and provide tips based on what they had learnt from their own experience as junior students. Interactions with the tutors have greatest impact when they provide adequate time to the learner [15]. Tutees felt that the tutors had taken the time to develop a rapport with them, which has been shown to assist the development of students’ autonomy and competence [23–25]. Students reported feeling comfortable with their tutors, and felt they were able to ask questions freely.

#### Significance of the study

Use of an existing theoretical framework may assist medical education researchers in understanding and determining the value of the presented research. According to McMillan, an educational research study should display consistency with established theory, building on what is already known [9]. By utilising the ExBL model as a theoretical lens to interpret and understand the data, we have strengthened our study. ExBL theory may provide a useful framework to explore affordance of such activities at other hospital based clinical schools.

#### Limitations of the study

It should be acknowledged that the students who participated in the PT program had voluntarily chosen to do so, which may have biased our results. Collection of qualitative data only through focus groups alone has some limitations, where students may not be completely open in the presence of their peers. However, we felt that by carrying out the focus groups for peer tutors and peer tutees separately, students would be more likely to speak freely about their experiences, whether positive or negative, hence limiting any adverse influences on the results of our study. We therefore feel that the conclusions we present in our study are valid.

## Conclusion

Participation in learning within medical curricula involves a socialisation process, to which our hospital based peer tutoring program offered affordance. Students’ learning processes were fostered with the PT program framework by organisational support, pedagogic support and affective support [10]. The social constructs of the PT program helped senior students to revise knowledge, develop their professional identity and recognise their significant roles and responsibilities as medical students and their future roles as medical practitioners with teaching and lifelong learning responsibilities [13]. Concurrently, junior students were provided with a valuable learning experience that proved qualitatively different to traditional teaching by faculty, and assisted their integration into the hospital environment [5].

## Abbreviations

CCS: Central Clinical School
ExBL: experienced based learning
PT: peer teaching
